# Assessing the preparedness and future-readiness of Malaysian community pharmacists in Klang Valley regarding the use of medical marijuana

**DOI:** 10.1186/s12913-024-11008-w

**Published:** 2024-04-25

**Authors:** Fu Wai Kuang, Muhammad Junaid Farrukh

**Affiliations:** Faculty of Pharmaceutical Sciences, UCSI University, Cheras, 56000 Kuala Lumpur, Malaysia

**Keywords:** Medical Marijuana, Legalisation, Knowledge, Attitude

## Abstract

**Background:**

This study investigated community pharmacists' level of knowledge and attitude towards medical marijuana and its association with sociodemographic characteristics.

**Methods:**

A cross-sectional study was conducted from 21 February 2022 to 15 November 2022. Community pharmacists working in Klang Valley were given a self-administered questionnaire. This survey instrument facilitated the collection of information about their sociodemographic attributes, training background, and knowledge and attitude concerning medical marijuana. Through rigorous analysis of the accumulated data, discernible factors correlating with the levels of knowledge and attitudes surrounding medical marijuana were identified.

**Results:**

The majority (*n*=149, 53.8%) of participants had low knowledge of medical marijuana. Participants with lower knowledge of medical marijuana tend to have a negative attitude toward medical marijuana. Besides that, male participants showed higher knowledge of medical marijuana than female participants. Furthermore, it was found that atheists had the most negative attitude among other religions toward medical marijuana.

**Conclusion:**

Most community pharmacists in Malaysia lack sufficient knowledge about medical marijuana. This indicates that Malaysian pharmacists are not future-ready and need to equip themselves with adequate knowledge of the indications and adverse effects of medical marijuana if it is to be legalised one day. Thus, there is a need for improved training and education of pharmacists around cannabis-based medicines.

**Supplementary Information:**

The online version contains supplementary material available at 10.1186/s12913-024-11008-w.

## Introduction

Marijuana is one of the most extensively utilised psychoactive substances. Nowadays, the use of marijuana is seen as it is associated with crime, recreational use and social problems [1]. Marijuana is used regularly by as many as 20 million individuals in the United States and Europe and millions more in other parts of the world, even though it is banned in most nations [2].

Similar to various other illicit substances, marijuana exerts an influence on dopamine (DA) transmission within the nucleus accumbent of the brain. This mechanism is hypothesised to underlie the pleasurable outcomes associated with drug use and the consequent neuroadaptive alterations contributing to addictive behaviour. Notably, investigations involving human subjects through neuroimaging techniques have revealed that illicit drug consumption induces an augmentation in dopamine (DA) release within the striatal region. These heightened dopaminergic responses have been associated with the subjective perception of reward [3].

These signs and symptoms might vary from person to person and can be moderate to severe. Although they might not be severe or hazardous, these symptoms can be uncomfortable. You were more likely to have withdrawal symptoms the longer you consumed marijuana [4].

Marijuana intoxication may cause many side effects. Many users say they have an insatiable hunger. Marijuana has a sedative, euphoric, and mildly relaxing impact on users. Marijuana smoking causes quick and predictable signs and symptoms. Effects from ingesting marijuana can be more gradual and occasionally unpredictable. These side effects were reduced short-term memory, dry mouth, diminished perception and motor skills, and red eyes [5]. Despite their bad reputation, if used appropriately, marijuana brings many benefits; these include lowering blood pressure, reducing inflammation, preventing relapse in drug and alcohol addiction, treating anxiety disorders, fighting cancer, preventing seizures and more [6].

More than 40 countries have legalised consumption of cannabis for medicinal purposes. There have been discussions about decriminalising marijuana in Malaysia. Decriminalising drugs does not mean that the drug will be legalised to use. Instead, it entails the retention of the drug's illegal status, albeit with a modification in the enforcement approach. Specifically, individuals found in possession of or engaged in the administration of the drug would not be subjected to the stringent legal repercussions that were previously enforced. Instead, alternate punitive measures such as fines, community service, or participation in drug treatment programs would be instituted. Consequently, the resultant punitive actions would be of a lesser severity than those stipulated by the preceding legal framework [7].

The Malaysian government had previously considered decriminalisation as a dual-policy approach. However, decriminalisation was more focused on drugs such as heroin and morphine, in which the primary administration method is through intravenous injection; this method of administration comes with high risks of transmitting HIV. In this case, people who inject these types of drugs were given free syringes through the National Syringe Exchange Programme (NSEP) or enrolment in the Methadone Replacement Therapy Programme (MRT) [8].

In Malaysia, marijuana consumption is regulated under the Dangerous Drugs Act (DDA) of 1952. Recent events like the push for the legalisation of medical marijuana in Thailand, which would be the first Asian country to legalise it [9], have added pressure on Malaysia to revisit its marijuana laws. Former Health Minister Khairy Jamaluddin asserted that the utilisation of cannabis-based medical products is authorised following compliance with prevailing laws, including the Dangerous Drugs Act 1952, Poisons Act 1952, and Sale of Drugs Act 1952 [10].

According to a systematic review on studies conducted in USA and Australia, it was found that pharmacists has low knowledge on medical marijuana and they perceived that they were underprepared when engaging with patients about medicinal cannabis [11]. Similar results were reported in studies done in Jordan where pharmacists had low knowledge of medical marijuana [12]. However, a study done in Thailand reported that most health care providers revealed that they had low to very low self-perceived knowledge about medical cannabis use (60–70%). However, for Item 6, adverse effects and warning signs and caution for patients in medical cannabis use, most had moderate self-perceived knowledge about medical cannabis use [13].

Little to no studies about marijuana have been done in Malaysia, especially among community pharmacists in Malaysia. Therefore, This study aimed to estimate the knowledge and attitude towards medical marijuana among community pharmacists in Klang Valley.

## Materials and Methods

### Study design

The study was a cross-sectional study conducted among community pharmacists in Klang Valley using a self-administered questionnaire.

### Study population

Registered community pharmacists here refer to pharmacists with Type A licenses from the Division of Pharmaceutical Service, Ministry of Health Malaysia, where a Type A license was defined as the license issued to a pharmacist to import, store, and deal with wholesale and retail.

### Sampling method and sample size

The complete sampling list was obtained from the list of registered Type A licenses from the Division of Pharmaceutical Service, Ministry of Health Malaysia [14], where a Type A license is defined as the license issued to a pharmacist to import, store and deal by wholesale and retail. 920 community pharmacists were working full-time in a community pharmacy within the Klang Valley [15, 16].

The sample size is calculated using Raosoft software by keeping a confidence interval of 95%, a margin of error of 5% and 50% of the response rate. A minimum of 277 respondents were required in this study. A stratified sampling approach was utilised, where community pharmacies were allocated to one of nine strata based on the districts in the Klang Valley. A proportionate number of community pharmacists within each stratum were recruited using simple random sampling.

#### Inclusion criteria

Community pharmacists working full-time in a community pharmacy within the Klang Valley.

#### Exclusion Criteria

Locum pharmacists, provisionally registered pharmacists (i.e. interns), pharmacists with “wholesale only” licenses, veterinary pharmacists, and those unwilling to complete the questionnaire.

### Study Tool (Questionnaire)

The questionnaire is pre-validated and adopted from a previous study and literature review [17–21] and consists of 3 domains: demographics, knowledge of medical marijuana, and attitude towards medical marijuana. There were nine questions on demographics and personal factors, 20 questions on knowledge regarding the therapeutic effects of medical marijuana, 20 questions on knowledge regarding the adverse effects of medical marijuana and 24 questions on Attitude about medical marijuana.

#### Scoring criteria

##### Knowledge

Each selection answered correctly was given 1 mark and 0 marks for every wrong answer (Min 0 and Max 20 marks). The total marks were classified into 2 categories. The mean of the total scores of knowledge was used to determine the midpoint of ‘good knowledge’ [22].

The FDA-approved analogs of marijuana include cannabidiol, dronabinol, and nabilone [23]. The assessment of the participant's knowledge of medical marijuana was based on its approved indications and adverse effects. The majority of states in the U.S. now allow for some form of medical marijuana. Each state has different regulations for medical marijuana and its availability to patients [24]. The approved indications were cancer, migraines, HIV, multiple sclerosis, Amyotrophic lateral sclerosis, Muscle spasms, Crohn's disease, epilepsy, Huntington's disease, Alzheimer's disease, PTSD and Hepatitis C. The non-approved indications include sleep apnea, Parkinson's disease, cystic fibrosis, vertigo, Tourette's disease, depression, hypertension and schizophrenia.

The correct adverse effects were memory impairment, hallucinations, worsening asthma, dizziness, blurred vision, anxiety, tachycardia, depression, nausea, birth defects, insomnia, seizures and stroke. The wrong adverse effects were water retention, muscle aches, constipation, cataracts, increased bleeding, anemia, and diabetes.

##### Attitude

This study used the Likert Scale to determine the attitude toward medical marijuana. 1 = Strongly Disagree, 2 = Disagree, 3 = Neutral, 4 = Agree, 5 = Strongly Agree. The total score was calculated, with a minimum score of 24 and a maximum score of 120. The higher score indicates a more positive attitude, and the lower score indicates a negative attitude toward medical marijuana. The mean of the total attitude scores was used to determine the midpoint of ‘positive attitude’ [22].

## Validation of questionnaire and pilot study

Content validity was done by five experts (physicians, academicians, and pharmacists). A pilot study was first carried out on 30 participants to ensure the reliability of the questionnaire formulated. Data collection was done using online mode with Google Forms. Internal consistency was calculated using Cronbach's alpha coefficient, which was 0.71.

The validated questionnaire is attached as a supplementary file.

## Data collection

Selected pharmacists were conveniently approached at their respective community pharmacies. Respondents were informed about the purpose of our research and invited to participate in this study. Before filling out the questionnaire, a written consent form and patient information sheet were attached to the questionnaire and distributed to the respondents.

## Data analysis

Statistical analyses were performed using the IBM SPSS Statistics Version 26. Data was analysed using descriptive and inferential analysis. For descriptive analysis, such as percentages, mean, and standard deviation were used to report demographic characteristics. Level of knowledge and attitude was presented as percentage, mean and SD. The mean difference in knowledge and attitude scores between sociodemographic characteristics was reported using the T-test and the ANOVA test based on the variables. The categorical association of knowledge and attitude was reported using the Chi-Square test.

## Results

A total of 277 respondents have agreed to participate in this survey. All the respondents were working as community pharmacists in Klang Valley, Malaysia. The majority of the participants (87.4%, *n*=242) hold a degree certificate while the rest (12.6%, *n*=35) have a Masters certificate. 65.7% (*n*=182) worked at a chain pharmacy, 20.6% (*n*=57) worked at a multi-outlet, and 13.7% (*n*=38) worked at a single outlet. 62.5% (*n*=173) of the participants had five or less working experience, while 37.5% (*n*=104) had more than five years of working experience.

The knowledge score ranged between 15 and 34 with a mean score of 24.24. Overall, 128 (46.2%) of participants have high knowledge and 149 (53.8%) of participants have low knowledge about medical marijuana. There was a significant difference (*p*<0.01) in mean knowledge scores between the two genders, where male participants had higher knowledge of medical marijuana than female participants. Moreover, The mean knowledge score was higher for those who graduated from public school than those who graduated from private school (*p*<0.01).

The knowledge of marijuana among individuals with less than five years of professional experience differed significantly (*p*<0.01) compared to those with more than five years of working experience.

Nearly half (*n*=130, 46.9%) of participants had a negative attitude towards medical marijuana. There was a significant difference in mean attitude scores between religion (*p*<0.01) and race (*p*=0.005). The post hoc test revealed that this difference was larger between atheists and Hindu religions. Regarding race, the most significant difference was between the Chinese and Indian races. The details of Sociodemographic characteristics concerning Knowledge and Attitude are shown in Table 1.

**Table 1:** Sociodemographic characteristics relation to Knowledge and Attitude toward medical marijuana

**Variables**	**Categories**	**Knowledge of Medical Marijuana**	***p*-value**	**Attitude towards Medical Marijuana**	***p*-value**
**Age**	20-29	1.80±0.59	0.139^b^	2.34±0.47	0.928^b^
	30-39	1.81±0.59		2.36±0.48	
	40-49	1.84±0.52		2.30±0.46	
	50-59	2.21±0.43		2.29±0.47	
	≥60	2.00±0.00		2.50±0.71	
**Gender**	Male	1.96±0.46	0.001^a**^	2.34±0.48	0.78^a^
	Female	1.79±0.60		2.33±0.47	
**Pharmacy School**	Private	1.81±0.60	0.002^a**^	2.35±0.48	0.07^a^
	Public	1.88±0.47		2.29±0.46	
**Religion**	Buddha	1.84±0.57	0.24^b^	2.34±0.47	0.001^b**^
	Islam	1.90±0.41		2.52±0.51	
	Hindu	1.58±0.69		2.67±0.49	
	Christian	1.76±0.64		2.17±0.38	
	Atheist	2.25±0.50		2.00±0.00	
**Race**	Chinese	1.83±0.58	0.66^b^	2.30±0.46	0.005^b**^
	Malay	1.90±0.41		2.52±0.51	
	Indian	1.73±0.80		2.60±0.51	
**District**	Sabak Bernam	-	0.11^b^	-	0.17^b^
	Hulu Selangor	2.38±0.52		2.50±0.53	
	Kuala Selangor	2.00±0.00		2.33±0.58	
	Gombak	2.00±0.77		2.27±0.47	
	Petaling	1.81±0.55		2.37±0.48	
	Hulu Langat	1.86±0.60		2.20±0.41	
	Klang	1.63±0.52		2.38±0.52	
	Kuala Langat	2±-		3±-	
	Sepang	1.67±0.58		2.43±0.51	
**Education Level**	Bachelor	1.83±0.58	0.99^a^	2.34±0.47	0.78^a^
	Master	1.83±0.57		2.31±0.47	
**Pharmacy Setting**	Single outlet	2.03±0.49	0.64^b^	2.32±0.47	0.91^b^
	Multi outlet	1.84±0.53		2.36±0.48	
	Chain pharmacy	1.79±0.60		2.33±0.47	
**Work Experience**	≤5	1.79±0.59	0.01^a**^	2.35±0.48	0.31^a^
	>5	1.89±0.54		2.32±0.47	

^**^indicates o<0.05 is significant

^a^independent t-test

^b^one-way ANOVA

**Fig. 1:**
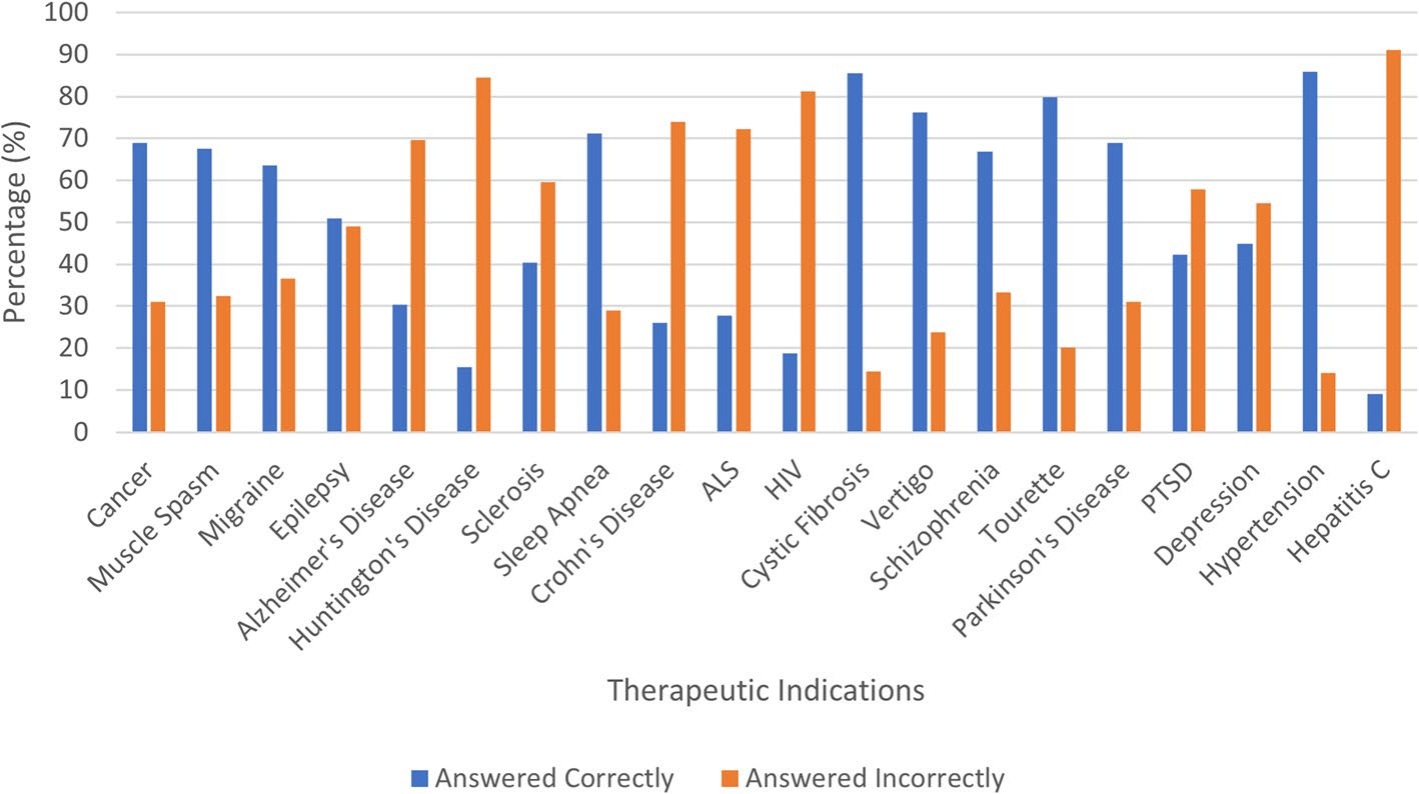
Knowledge of therapeutic effects regarding medical marijuana of respondent

The participants' responses regarding their knowledge of the therapeutic effects of medical marijuana can be found in Fig. 1.

**Fig. 2:**
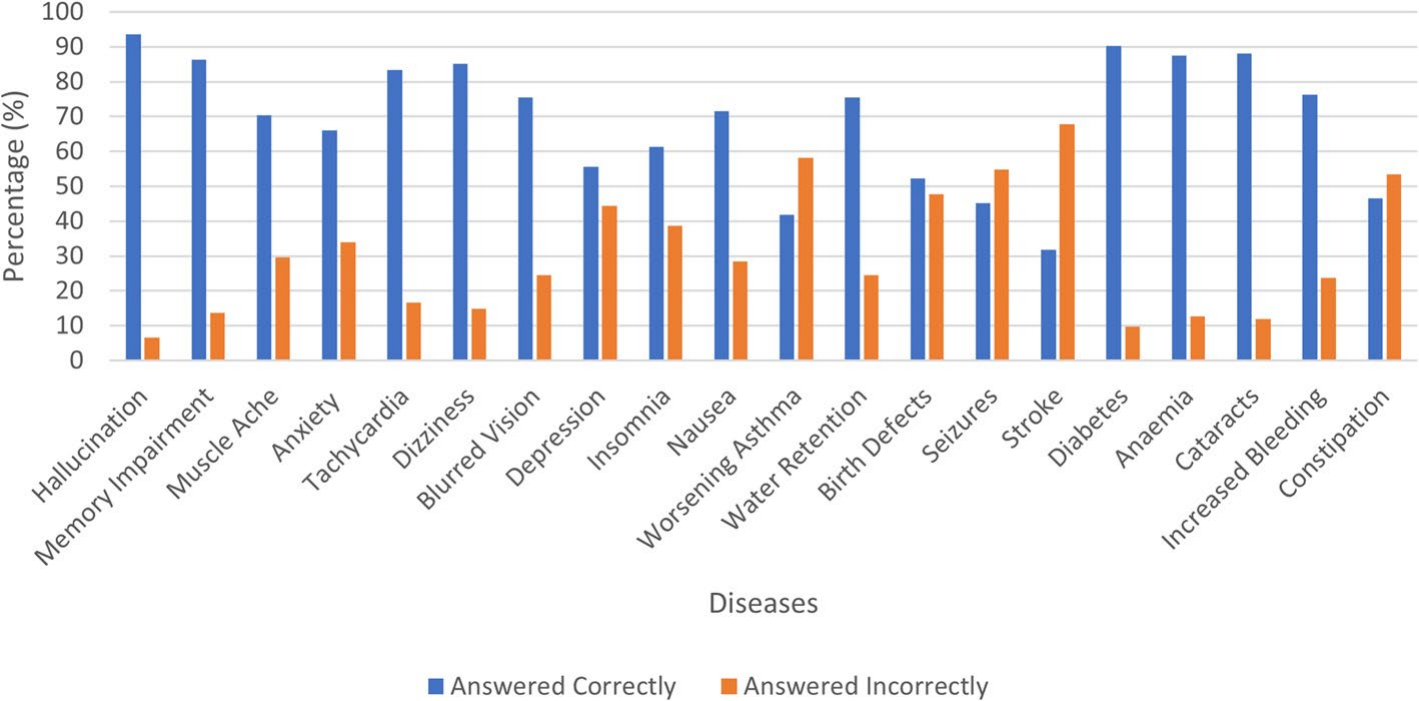
Knowledge of adverse effects regarding medical marijuana of respondent

Overall, 157 (56.7%) of the participants showed good knowledge about the adverse effects of medical marijuana while 120 (43.3%) of participants showed poor knowledge about the adverse effects of medical marijuana. Most participants (93.5%, *n*=259) chose hallucination as an adverse effect of using medical marijuana while only 9.7% (*n*=27) chose diabetes as a side effect, which was a wrong answer. Participant’s response to knowledge regarding the adverse effects of medical marijuana is shown in Fig. 2.

It was found that nearly half (*n*=130, 46.9%) of the participants had a negative attitude towards medical marijuana. 45.1% (*n*=125) of participants chose neutral when asked whether medical marijuana should be legalised for medicinal use. Regarding the safety of medical marijuana, 67.5% (*n*=182) agreed that it was safe as long as it was used responsibly for therapeutic use. However, the results also showed some concern over medical marijuana. 57% (*n*=158) chose to agree about the safety of medical marijuana, and 55.6% (*n*=154) of participants were concerned about the consistency in the quality of medical marijuana. Overall, none of the participants chose strongly to disagree regarding whether they were comfortable talking about medical marijuana. More details can be found in Table 2.

**Table 2:** Attitude about medical marijuana

Question	Strongly Disagree n(%)	Disagree n(%)	Neutral n(%)	Agree n(%)	Strongly Agree n(%)
Medical marijuana should be legalised for medicinal use.	11 (4%)	32 (11.6%)	125 (45.1%)	85 (30.7%)	24 (8.7%)
Marijuana is safe when used responsibly for medicinal use.	3 (1.1%)	13 (4.7%)	45 (16.2%)	182 (65.7%)	34 (12.3%)
Legalising medical marijuana will increase crime rates.	8 (2.9%)	35 (12.6%)	94 (33.9%)	113 (40.8%)	27 (9.7%)
Legalising medical marijuana will hurt the “war on drugs” effort.	7 (2.5%)	32 (11.6%)	155 (56.%)	67 (24.2%)	16 (5.8%)
Most people who support medical marijuana legalisation are drug abusers.	36 (13%)	126 (45.5%)	84 (30.3%)	26 (9.4%)	5 (1.8%)
I am concerned about the safety of medical marijuana.	4 (1.4%)	15 (5.4%)	59 (21.3%)	158 (57%)	41 (14.8%)
I am concerned about the consistency in the quality of medical marijuana.	2 (0.7%)	13 (4.7%)	55 (19.9%)	154 (55.6%)	53 (19.1%)
I am concerned about the regulations regarding medical marijuana.	3 (1.1%)	3 (1.1%)	36 (13%)	147 (53.1%)	88 (31.8%)
I am concerned that there is a potential addiction to marijuana use.	2 (0.7%)	8 (2.9%)	33 (11.9%)	137 (49.5%)	97 (35%)
I am concerned that there is limited evidence of the therapeutic benefits of medical marijuana.	8 (2.9%)	38 (13.7%)	92 (33.2%)	119 (43%)	20 (7.2%)
People usually have a good time when using marijuana.	2 (0.7%)	46 (16.6%)	143 (51.7%)	73 (26.4%)	13 (4.7%)
Marijuana is a dangerous drug.	9 (3.2%)	37 (13.4%)	128 (46.2%)	83 (30%)	20 (7.2%)
I would be concerned if friends or family were using medical marijuana.	9 (3.2%)	43 (15.5%)	64 (23.1%)	115 (41.5%)	46 (16.6%)
I would be willing to use medical marijuana if prescribed.	12 (4.3%)	29 (10.5%)	106 (38.3%)	115 (41.5%)	15 (5.4%)
Medical marijuana may result in dependence.	2 (0.7%)	16 (5.8%)	78 (28.2%)	154 (55.6%)	27 (9.7%)
Medical marijuana will act as a gateway drug to the use of other illicit drugs.	3 (1.1%)	37 (13.4%)	95 (34.3%)	114 (41.2%)	28 (10.1%)
The benefits of using medical marijuana outweigh the harms and risks associated.	3 (1.1%)	42 (15.2%)	156 (56.3%)	70 (25.3%)	6 (2.2%)
The use of medical marijuana will lead to marginalisation by society.	4 (1.4%)	39 (14.1%)	149 (53.8%)	78 (28.2%)	7 (2.5%)
I am familiar with the current laws and regulations regarding medical marijuana in Malaysia.	21 (7.6%)	101 (36.5%)	100 (36.1%)	45 (16.2%)	10 (3.6%)
The use of medical marijuana can lead to recreational use and eventually lead to abuse.	4 (1.4%)	35 (12.6%)	69 (24.9%)	146 (52.7%)	23 (8.3%)
Dispensing marijuana and its derivatives in pharmacies would expose the pharmacies to specific dangers (robbery, insisting on prescribing without prescription, etc…)	2 (0.7%)	23 (8.3%)	52 (18.8%)	137 (49.5%)	63 (22.7%)
A doctor’s prescription is mandatory for dispensing medical marijuana and its derivatives.	2 (0.7%)	4 (1.4%)	28 (10.1%)	86 (31%)	157 (56.7%)
The use of medical marijuana and its derivatives is justified in the case of terminally ill patients.	1 (0.4%)	7 (2.5%)	45 (16.2%)	125 (45.1%)	99 (35.7%)
I feel comfortable discussing medical marijuana.	0 (0%)	8 (2.9%)	82 (29.6%)	134 (48.4%)	53 (19.1%)

There was no significant association between knowledge and attitude. However, Participants with low knowledge were more likely to have a negative attitude, as shown in Table 3

**Table 3:** Association between knowledge and attitude of medical marijuana

		**Attitude regarding Medical Marijuana, n (%)**			***p*-value**
		**Positive**	**Negative**	**Total**	
**Knowledge of Medical Marijuana, n (%)**	**High**	73 (26.35)	55 (19.86)	128 (46.21)	0.22^a^
	**Low**	74 (26.71)	75 (27.08)	149 (53.79)	
Total		147 (53.07)	130 (46.93)	277 (100)	

^a^Chi-square test

## Discussion

This study aimed to estimate the knowledge and attitude towards medical marijuana among community pharmacists in Klang Valley.

Most of the participants in this survey had poor knowledge of medical marijuana. Although there no such study done among pharmacists in Malaysia, however, A similar study in Melaka targeting medical students showed that the participants had a low knowledge of medical marijuana [25]. Another study reported that pharmacists from Minnesota, United States, had a low level of knowledge regarding medical marijuana [26]. One plausible explanation could be rooted in the limited inclusion of medical marijuana within the Malaysian pharmacy curriculum. Consequently, pharmacists may lack comprehensive education on this subject. Additionally, reliance on online resources for learning about medical cannabis may contribute to potential inaccuracies or gaps in knowledge acquisition [24].

An observed correlation was established between participant gender and their level of comprehension regarding medical marijuana, wherein male participants exhibited a higher degree of knowledge concerning medical marijuana in comparison to their female counterparts. The same conclusion was drawn from a study in Australia with university students, which found that males were more confident concerning their knowledge of cannabis than females [27]. This could be because males tend to interact more with such substances or have friends or relatives who know about marijuana [28].

The data analysis shows that different ages have different attitudes towards medical marijuana. Another study in Michigan done with healthcare-related workers observed that younger participants were more likely to accept the decriminalisation of medical marijuana [29]. The older population were found to be unsure about the legalisation of medical marijuana. This result, however, contradicts our findings, which suggested that age was not a significant factor in the acceptance towards the decriminalisation of medical marijuana.

Religion and race were also found to have a significant association with the attitude toward medical marijuana. The research revealed that Hindus/Indians exhibit the highest level of acceptance towards medical marijuana in comparison to followers of other religions. The outcome aligns with expectations, given the association of marijuana with Shiva, a prominent Hindu deity. According to religious rites, cannabis is believed to cleanse sins, unite one with Shiva and avoid the miseries of hell in the future life [30].

## Limitations

Data was collected using convenience sampling methodology, a sort of non-probability sampling approach in which the samples were chosen from a group of people who are accessible or easy to come into touch with [31].

Furthermore, the study was conducted in Klang Valley areas as more pharmacies are available. As a result, our study did not reach the outskirts of this area. Less populated areas were also excluded as the area was too far to be conducted. To mitigate potential biases, the study should be conducted comprehensively throughout Malaysia.

## Conclusion

Community pharmacists in Klang Valley were mainly observed to have low knowledge of medical marijuana. This may be due to a lack of education regarding medical marijuana. This indicates that Malaysian pharmacists need to equip themselves with more knowledge of medical marijuana if medical marijuana were to be legalised in the future.

### Supplementary Information

**Supplementary Material 1.** 

## Data Availability

Data will be available upon request.
