# Supplementary material for: Assessing the preparedness and future-readiness of Malaysian community pharmacists in Klang Valley regarding the use of medical marijuana
Source: BMC Health Serv Res. 2024 Apr 25;24:524. doi: 10.1186/s12913-024-11008-w (PMC11044563; doi:10.1186/s12913-024-11008-w)
Supplement: Supplementary file 1 — Supplementary Material 1. [file 12913_2024_11008_MOESM1_ESM.docx]

Appendix:

Questionnaire

| 1. Demographics and Personal Factors | |
| --- | --- |
| 1. What is your age? | _______ Years |
| 1. What is your gender? | 1. Male 2. Female |
| 1. Is your pharmacy school a private or public institution? | 1. Private 2. Public |
| 1. What is your religion? | 1. Buddha 2. Islam 3. Hindu 4. Others (____________) |
| 1. What is your race? | 1. Chinese 2. Malay 3. Indian 4. Others (_____________) |
| 1. Which district are you currently working at? | 1. Sabak Bernam 2. Hulu Selangor 3. Kuala Selangor 4. Gombak 5. Petaling 6. Hulu Langat 7. Klang 8. Kuala Langat 9. Sepang 10. Others (_____________) |
| 1. What is your highest education level? | 1. Bachelors 2. Masters 3. PhD |
| 1. Pharmacy setting | 1. Single outlet 2. Multi outlet 3. Chain pharmacy |
| 1. Working experience as a community pharmacist | _____________ years |

*B) (i) Knowledge regarding the therapeutic effects of medical marijuana.*

| **Therapeutic Indication** | **Response** |
| --- | --- |
| Cancer  Muscle spasm  Migraine  Epilepsy  Alzheimer’s Disease  Huntington’s Disease  Multiple Sclerosis  Sleep Apnea  Crohn’s Disease  Amyotrophic Lateral Sclerosis  HIV  Cystic Fibrosis  Vertigo  Schizophrenia  Tourette’s Disease  Parkinson’s Disease  PTSD  Depression  Hypertension  Hepatitis C | Y / N  Y / N  Y / N  Y / N  Y / N  Y / N  Y / N  Y / N  Y / N  Y / N  Y / N  Y / N  Y / N  Y / N  Y / N  Y / N  Y / N  Y / N  Y / N  Y / N |

***B (ii)*** *Knowledge regarding adverse effects of medical marijuana.*

| **Adverse Effects** | **Response** |
| --- | --- |
| Hallucination  Memory Impairment  Muscle Ache  Anxiety  Tachycardia  Dizziness  Blurred Vision  Depression  Insomnia  Nausea  Worsening Asthma  Water Retention  Birth Defects  Seizures  Stroke  Diabetes  Anaemia  Cataracts  Increased Bleeding  Constipation | Y / N  Y / N  Y / N  Y / N  Y / N  Y / N  Y / N  Y / N  Y / N  Y / N  Y / N  Y / N  Y / N  Y / N  Y / N  Y / N  Y / N  Y / N  Y / N  Y / N |

*C) Attitude about medical marijuana*

| Items | Strongly Disagree | Disagree | Neutral | Agree | Strongly Agree |
| --- | --- | --- | --- | --- | --- |
| Medical marijuana should be legalized for medicinal used. |  |  |  |  |  |
| Marijuana is safe when used responsibly for medicinal used. |  |  |  |  |  |
| Legalizing medical marijuana will increase crime rates. |  |  |  |  |  |
| Legalizing medical marijuana will hurt the “war on drugs” effort. |  |  |  |  |  |
| Most people who support medical marijuana legalization are drug abusers. |  |  |  |  |  |
| I am concerned about the safety of medical marijuana. |  |  |  |  |  |
| I am concerned about the consistency in the quality of medical marijuana. |  |  |  |  |  |
| I am concerned about the regulations regarding medical marijuana. |  |  |  |  |  |
| I am concerned that there is a potential addiction to marijuana use. |  |  |  |  |  |
| I am concerned that there is limited evidence of the therapeutic benefits of medical marijuana. |  |  |  |  |  |
| People usually have a good time when using marijuana. |  |  |  |  |  |
| Marijuana is a dangerous drug. |  |  |  |  |  |
| I would be concerned if friends or family were using medical marijuana. |  |  |  |  |  |
| I would be willing to use medical marijuana if prescribed. |  |  |  |  |  |
| Medical marijuana may result in dependence. |  |  |  |  |  |
| Medical marijuana will act as a gateway drug to the use of other illicit drugs. |  |  |  |  |  |
| Benefits of using medical marijuana outweigh the harms and risk associated. |  |  |  |  |  |
| The use of medical marijuana will lead to marginalization by society. |  |  |  |  |  |
| I am familiar with the current laws and regulations regarding medical marijuana in Malaysia. |  |  |  |  |  |
| The use of medical marijuana can lead to recreational use and eventually lead to abuse. |  |  |  |  |  |
| Dispensing cannabis and its derivatives in pharmacies would expose the pharmacies to certain dangers (robbery, insisting on prescribing without prescription etc…) |  |  |  |  |  |
| Doctor’s prescription is mandatory for dispensing medical marijuana and its derivatives. |  |  |  |  |  |
| The use of medical marijuana and its derivatives is justified in the case of terminally ill patients. |  |  |  |  |  |
| I feel comfortable discussing about medical marijuana. |  |  |  |  |  |
